# The meaning of the social support network for women in situations of violence and breastfeeding*

**DOI:** 10.1590/1518-8345.3313.3316

**Published:** 2020-07-15

**Authors:** Nayara Girardi Baraldi, Angelina Lettiere-Viana, Diene Monique Carlos, Natália Rejane Salim, Daniela Taysa Rodrigues Pimentel, Juliana Stefanello

**Affiliations:** 1 Universidade de São Paulo, Escola de Artes, Ciências e Humanidades, São Paulo, SP, Brazil.; 2 Scholarship holder at the Coordenação de Aperfeiçoamento de Pessoal de Nível Superior (CAPES), Brazil.; 3 Universidade de São Paulo, Escola de Enfermagem de Ribeirão Preto, PAHO/WHO Collaborating Centre at the Nursing Research Development, Ribeirão Preto, SP, Brazil.; 4 Universidade Federal de São Carlos, Departamento de Enfermagem, São Carlos, SP, Brazil.

**Keywords:** Social Networking, Breast Feeding, Intimate Partner Violence, Violence Against Women, Health Personnel, Qualitative Research, Rede Social, Aleitamento Materno, Violência por Parceiro Íntimo, Violência contra a Mulher, Pessoal de Saúde, Pesquisa Qualitativa, Red Social, Lactancia Materna, Violencia de Pareja, Violencia contra la Mujer, Personal de Salud, Investigación Cualitativa

## Abstract

**Objective:**

to understand the meanings attributed to the social support network of women breastfeeding and in situations of violence by an intimate partner.

**Method:**

a qualitative study, carried out with 21 women, through semi-structured interviews and data analyzed by the Method of Interpretation of the Senses in the light of the conceptual framework of Social Support Network.

**Results:**

all women suffered violence by the partner in the puerperium and only one of them maintained exclusive breastfeeding until 180 days postpartum. In the analysis, the category entitled “The action of the social support network in the face of breastfeeding in the context of intimate partner violence” emerged, with two subcategories: “Interpersonal support network” and “Institutional support network”. In the interpersonal network, the partner was little mentioned, on the other hand, there was a greater participation of other women. In the institutional network, non-resolution and actions centered on biological character were evident.

**Conclusions:**

the search for help in the interpersonal network stood out in comparison to the institutional network, both with regard to the issue of violence and breastfeeding and the actions related to it, mostly ineffective, characterized by counseling and referrals.

## Abstract (pt)

**Objetivo:**

compreender os significados atribuídos à rede de suporte social de mulheres em aleitamento materno e em situação de violência por parceiro íntimo.

**Método:**

estudo qualitativo, realizado com 21 mulheres, por meio de entrevistas semiestruturadas e de dados analisados pelo Método da Interpretação dos Sentidos à luz do referencial conceitual de Rede de Suporte Social.

**Resultados:**

todas as mulheres sofreram violência pelo parceiro no puerpério e apenas uma delas manteve o aleitamento materno exclusivo até 180 dias pós-parto. Na análise, emergiu a categoria intitulada “A ação da rede de suporte social diante do aleitamento materno no contexto de violência por parceiro íntimo”, com duas subcategorias: “Rede de suporte interpessoal” e “Rede de suporte institucional”. Na rede interpessoal, o companheiro foi pouco referido, em contrapartida, houve maior participação de outras mulheres. Na rede institucional, ficaram evidentes a não resolutividade e as ações centradas no caráter biológico.

**Conclusões:**

a busca por ajuda na rede interpessoal sobressaiu-se em comparação à rede institucional, tanto no que se refere à questão da violência quanto à amamentação e às ações a ela relacionadas, em sua maior parte, pouco efetivas, caracterizadas por aconselhamentos e encaminhamentos.

## Abstract (es)

**Objetivo:**

comprender los significados atribuidos a la red de apoyo social de las mujeres que amamantan y atraviesan situaciones de violencia por parte de una pareja íntima.

**Método:**

estudio cualitativo, realizado con 21 mujeres, a través de entrevistas semiestructuradas y datos analizados por el Método de Interpretación de los Sentidos a la luz del marco conceptual de la Red de Apoyo Social.

**Resultados:**

todas las mujeres sufrieron violencia por parte de la pareja en el puerperio y solo una de ellas mantuvo la lactancia materna exclusiva hasta 180 días después del parto. En el análisis, emergió la categoría titulada “La acción de la red de apoyo social frente a la lactancia materna en el contexto de la violencia por parte de la pareja íntima”, con dos subcategorías: “Red de apoyo interpersonal” y “Red de apoyo institucional”. En la red interpersonal, la pareja fue poco mencionada, y, por otro lado, hubo una mayor participación de otras mujeres. En la red institucional, la falta de resolución y las acciones centradas en el carácter biológico fueron evidentes.

**Conclusiones:**

se destacó la búsqueda de ayuda en la red interpersonal si se la compara con la red institucional, tanto en relación al tema de la violencia como a la lactancia materna y/o acciones a ella relacionadas, en su mayoría ineficaces, caracterizadas por protocolos y derivaciones.

## Introduction

In almost all countries, 80% of newborns receive breast milk (BM) at birth. However, most of these nations have rates of less than 50% of exclusive breastfeeding (EBF) until the sixth month of life and, even after international and national efforts, the rate remains below that recommended by the World Health Organization (WHO) and United Nations Children’s Fund (Unicef)^(^ 1 ^-^ 2 ^)^.

Until 2006, Brazil had an upward trend in breastfeeding (BF) rates and, since then, has maintained stabilization, although breastfeeding indicators have identified that only 36.6% of children remained in EBF until the sixth month of life, as it corroborates rates in underdeveloped and developing countries^(^ 3 ^-^ 4 ^)^. To broaden the understanding of such indicators, the complexity of the breastfeeding phenomenon must be considered, since it goes beyond biological aspects and is related to historical, cultural, psychological and social factors, so that these social determinants can be facilitators for the early weaning^(^ 5 ^)^.

Thus, it is important to understand that breastfeeding is not under the woman’s exclusive responsibility, but also a collective duty^(^ 5 ^)^. In this sense, the social support network (SSN), made up by a partner, family, civil society, State and public institutions, such as the health sector, has a significant role in the experience of healthy breastfeeding for women^(^ 6 ^)^, as well as for the maintenance of this practice.

The SSN can be defined as the set of interpersonal and social relationships^(^ 7 ^)^, which can play both a protective and a risky role for individuals, depending on the context in which these relationships develop^(^ 8 ^)^. Consequently, the relationships established in the SSN are associated with the social, cultural, political and religious contexts that are integrated among the generations and, in this sense, directly interfere in the way breastfeeding occurs in the live of women^(^ 9 ^)^.

That said, the importance of incorporating, in the care practices, the participation of the SSN in breastfeeding situations, in order to identify and satisfy the needs of women, as well as minimize doubts, anxieties and ambiguous ideas generated by social practices (of the family support) and scientific knowledge (health field) in order to establish a pleasant breastfeeding^(^ 10 ^)^. When analyzed the experiences of women in the act of breastfeeding and their interface with the SSN, in interpersonal and institutional aspects, a question echoes: How is the SSN of women who experience breastfeeding and, concomitantly, the situation of intimate partner violence (IPV) configured?

The proposition of the IPV context is justified by two questions: First, it is evident that the SSN is reduced and fragmented in the context of violence^(^ 11 ^)^; nevertheless, it appears that the IPV is related to unfavorable breastfeeding practices, such as a low propensity to start breastfeeding, a lower desire to breastfeed, a low probability of maintaining breastfeeding and a greater chance of weaning early^(^ 12 ^)^.

Furthermore, this study is justified by the literary gap on the influence of the SSN in these two concomitant conditions, that is, in BF practice in women who experience the IPV. Pioneering study on and breastfeeding self-efficacy shows that the association between both phenomena exposes women to unfavorable conditions to breastfeed and reinforces the importance of training health professionals in order to understand and work with this problem^(^ 13 ^)^. In Brazil, a single study was dedicated to the theme and observed that the lack of an SSN, associated with an IPV context, can be an obstacle to BF practice^(^ 14 ^)^. Therefore, this study aimed to understand the meanings attributed to the social support network of women breastfeeding and in situations of violence by an intimate partner.

## Method

Qualitative study carried out in a city in the inland of the state of São Paulo. 21 women participated in this study, key informants^(^ 15 ^)^, which were selected from a cross-sectional study carried out with 315 women who received childbirth assistance in a public maternity hospital at usual risk. The research identified the prevalence of IPV cases before pregnancy and in the pregnancy-puerperal cycle. Thus, the inclusion criteria for women in this study were: Having participated in the transversal project; residing in the research municipality; being primiparous; having started breastfeeding; having experienced IPV in the puerperium; and having at least 180 days postpartum. At least 180 days postpartum were waited to access the EBF phenomenon^(^ 1 ^)^.

After the stage of identification and selection of participants, recruitment was carried out by invitation, via telephone, to participate in the study. There were no refusals to participate. The meeting was scheduled, according to the person’s availability in relation to the day, time and place. As places to conduct the interview, the Basic Health Unit, a private room at the university and the puerperal woman’s residence were made available. When the interview took place at the residence, two authors of the research traveled to the place, for safety reasons. The day before the interview, the first author, responsible for the research, confirmed the appointment by phone. Food, water and toys were made available on the spot for the interview to take place smoothly.

Data collection started in April 2015, ending in October of the same year, and was preceded by a pilot study for adequacy. For the interviews, a digital voice recorder was used. The interview took place only once, with an average duration of 42 minutes, and followed a semi-structured script, composed of the following guiding questions: “I would like you to tell me about the discussions between you and your partner and if you sought any help to face them. If so, how was that help? How was it to breastfeed in the face of these situations of fights and disagreements?” The total number of participants was determined by the aspects present in the statements, which began to be repeated and deepened to understand the meanings attributed to the SSN of the participants^(^ 16 ^)^.

Given the context of violence, leaflets and booklets with content on violence against women (VAW) and on the SSN in the municipality were offered to all women. On the occasion, these women were referred to the psychology service of a public university and, when required, they were also referred to the assistance support service for people in situations of violence or in risky conditions in the municipality.

In the analysis of the data, the interviews were fully transcribed by the first author, without the grammatical correction of their speeches being carried out, in order to maintain the original meaning. The data were analyzed using the method of interpretation of the senses, with the following steps: comprehensive reading of the collected material, exploration of the material and elaboration of the interpretative synthesis. Comprehensive reading seeks the particularities and the view of the material as a whole; in this stage, the units of meaning are established for further categorization, as shown in Figure 1. Exploration seeks to go beyond the text to bring up the subjective or what is implicit in the report and, in this way, to seek the meanings in the dialogue with the theoretical references, thus starting the inference. The interpretative synthesis is the moment of the interpretation to be worked according to the objectives of the study, with the theoretical references and with the empirical data^(^ 17 ^)^. In this perspective, the conceptual framework used was the Social Support Network^(^ 18 ^)^.

**Figure 1:** Sense units with the initial and intermediate codes for the construction of the final thematic subcategories. Ribeirão Preto, SP, Brazil, 2015

Initial codes	Intermediate codes	Final themes: Sub-category
Lack of partner support	The composition of the network according to the actions before the events: Breastfeeding and intimate partner violence Network interventions and interferences in the breastfeeding process Network interventions and interference in the face of intimate partner violence Past over care between generations and the bond	Network for interpersonal support
Violent actions of the partner and the reflexes on breastfeeding		
Support in the pregnancy-puerperal cycle of friends, cousins, sisters, mother and grandparents		
Advice and opinion from family and friends on breastfeeding		
Action by family members, friends or neighbors in the face of violence		
Reflections of violence on the network that tries to protect women		
Weakened relationships with people in the network due to intimate partner violence		
Network involvement in the face of intimate partner violence		
Network participation in the breastfeeding process		
Demotivation in breastfeeding due to intergenerational action		
Early feeding introduction and weaning		
Search for hospital service	Search for care in the institutional network Defragmentation and impersonality of care generated by the institutional network	Institutional support network
**Search for legal service**		
Search for the Women’s Police Station		
The Maria da Penha law does not exist		
Lack of police support		
Lack of correct information between services		
The joint ‘forgetfulness’		
Lack of individuality in care		
Fear of leaving the hospital		
Hospital routines and protocols for breastfeeding and/or formula		

To guarantee anonymity, the interviewees were coded by the word ‘Participant’, followed by Arabic numerals from one to 21 corresponding to the order in which the interviews were carried out. As for ethical aspects, the rules for research with human beings established in Resolution No. 466/12 of the National Health Council were followed. The study received approval from the Research Ethics Committee of the Ribeirão Preto College of Nursing, University of São Paulo, under the CAAE protocol: 24049713.4/0000.5393 were observed.

## Results

The participants were between 18 and 36 years old. Eight of them declared themselves white; seven, brown; five, black; and one, yellow. Eight completed high school; three, elementary education; and one finished higher education. With regard to occupation, 12 were housewives, five worked with a formal contract, two were unemployed, and one worked without a formal contract and another was a student.

Of the 21 participants, nine claimed to have suffered some type of IPV during pregnancy: Five psychological violence, one sexual violence and three associated psychological and physical violence. In the postpartum period, five suffered a single type of violence, while the others experienced two or more associated types. Regarding BF, of the 21 participants, only one breastfed exclusively until the sixth month. Overall, at the end of 180 days postpartum, seven babies were already weaned, while 13 were in breastfeeding with complementary fluids or porridge.

In the analysis of the empirical material of the interviews, a category was constructed entitled “The action of the social support network in the face of breastfeeding in the context of intimate partner violence”, which encompasses the meaning attributed by women who started BF in a context of IPV in relation to the composition and functionality of the network. This category was divided into two subcategories, which showed the phenomenon of IPV and BF according to the composition of the network, so they were entitled: “Interpersonal support network” and “Institutional support network”.

The first subcategory refers to the interpersonal support network and revealed that the partner was not part of this network, a fact aggravated by situations of abuse and violence: [...] *in the ward, I didn’t want to be discharged from the hospital, I was terrified of going home, I was afraid ... I dunno... with the belly like that, it hurt too much and it doesn’t give any work and with A. (partner) I couldn’t count and trust him to help me* [...] *(Participant 15); In addition, companion presented violent behavior when the woman breastfed:* [...] *If B. (baby) cried, he (partner) would say: ‘Wow, give this girl the breast’. Then it was no longer with affection, it was screaming!* [...] *when I got angry at the discussion, I thought: ‘Oh, get this girl out of here’.* [...] *(Participant 13).*

Most interpersonal networks were composed of other women, such as mothers, mothers-in-law, cousins, aunts and friends: *I expected more by the father* [...] *he was not the one who stayed there with the child, it was me* [...] *I was the one who supported everything and so all that he (baby) has today was me and my mother who arranged it, you know? (Participant 18).*

However, some participants reported little contact with family members, especially due to some relational incompatibility: [...] *I love my mother, we just don’t have that understanding, because as my father and my mother are separating, now she and I have moved further away because when I go there, she doesn’t take care of V. (daughter), she doesn’t help me, she doesn’t even say hi to me sometimes* [...] *I have no one to talk to... I only talk to you, I never went to open it to anyone, only God who knows everything, but only you, and my mother I never talked to talk about* [...] *(Participant 5).*

With regard to functionality, that is, the actions of the network in relation to IPV, the participants cited advice, words of support and help when they needed to be housed in the home of relatives: [...] *She (mother-in-law) goes to the house and talks and she says that he is very good to me* [...] *Just for them (relatives) that I told, because when I got to fight with him, I would go to their house and sleep there with them one day, two days* [...] *(Participant 8);* [...] *I arrived at his house (father), let’s suppose, with a mark on the arm, then he already knew, ‘it was S. who beat you‘. Get off him, he’s not a man for you. Go back to school, go look for a course, your father is here ... father may help you ...’, understood?!* [...] *L. (mother-in-law) also* [...] *she also always gave me good advices.* [...] *my family revolted at the same time with him, more with me, because like that, I fought with him, it wouldn’t be two or three weeks, I already wanted to go back with him, you know?!* [...] *(Participant 19).*

However, the interpersonal network itself contributed, indirectly, for the woman to remain and endure the situation of violence: [...] *I started to have an illusion in my head, a really family vision because my own mother says: “Since you have a daughter, it doesn’t hurt to try, right ...?”* [...] *(Participant 15).*

As for the network’s actions in the face of BF, it was found that family members, both of the woman and the partner, as well as friends and neighbors, were present, especially in the initial difficulties with BF: [...] *my mother-in-law helped me with everything, after LA was born she who helped me to look, I who breastfed her at dawn, so that I could doze off, she who kept looking* [...] *changed the diaper, made her sleep, went back to sleep some more and she looked* [...] *she helped me a lot, told me to keep sunbathing, it helped, you know, there’s a little pomade until my father-in-law bought it* [...] *(Participant 21).*

It is observed, in the statements, that the intergenerational actions of women with experience of motherhood and breastfeeding experience, especially mothers, were present in BF practice: [...] *I was discouraged to breastfeed my daughter because of my mother, understood?!* [...] *she (mother) tells us today that we suckled us up to a month old at most ... she said: ‘Oh, haven’t you tired of breastfeeding her yet? Wow, it’s time for you to get her off the breast... it’s time for you to feed this girl with baby pap* [...] [...] *I thought: ‘Wow, is it time for me to take her out?’* [...] *(Participant 19);* [...] *since being four months old, she (mother) started giving a little banana, took the apple broth and already gave, then, my mother already started because she (mother) said she wanted a fat baby* [...] *She (mother) always said: ‘That I’ve had three children, right, so you can give this, that, that other...* [...] *(Participant 2).*

The second subcategory refers to the institutional support network, that is, the services required by the participants. Regarding the composition of the institutional network, the institutions required were health services, judicial institutions and non-governmental organizations (NGOs). In terms of functionality, the institutional network was fragmented in relation to IPV: [...] *I went to the woman’s police station, then I made a Police Report against him with Maria da Penha law, that he had to stay 500 meters away from here and I also looked for the Women’s Coordination, there they told me to look for a lawyer* [... ] *(Participant 9).*

In addition to the woman’s pilgrimage, the lack of resolution and the intersectionality of the institutional network were evident: [...] *the girl who was there, in the small room, said to me: ‘Hi, this law Maria da Penha doesn’t exist!’, she said it in my face, and she said: ‘I’m sorry to tell you, but it doesn’t exist, the best thing you have is to heat some oil and throw it in his face. There is no such thing, I just give you advice, but if you want to go, you go to Cuiabá and take a corpus delicti exam and such, such, and such...’. I took it the other day, limping, with a black eye, I went to Cuiabá, arriving there I had to go to the Secretary of Health to do I don’t know what, they didn’t even know how to inform me, then I said: ‘That is why many women give up on Maria da Penha, because it is very complicated and nobody knows how to give the right information.’* [...] *(Participant 2).*

For this group of women, the health sector acted both for IPV and for breastfeeding care. However, the actions were based on biological care, in addition to not accepting the complaints of the participants: [...] *I had never had a child, so I think so, there was a lack of guidance at the hospital* [...] *around 2 a.m., she (health professional) took the girl there. They (health professionals) came back in the room at about 7 am* [...] *she (nurse) put the girl suckling out a lot of blood in the girl’s mouth [...]then they started giving milk to the baby, Nestogeno® or NAN®, only they came with 30 ml and we had to wait three hours* [...] *she spent two hours crying. And they said it was normal, that it could only happen after three hours.* [...] *(Participant 20);* [...] *the doctors said I didn’t have a lot of milk because of the nervousness I was going through, so she took too long to get the nipple at M. (maternity hospital), then the nurses came and tried to put her on the breast and it got to hurt too much* [...] *(Participant 8).*

## Discussion

The characteristics of the participants in this research corroborate the literature in the area. Reflecting on such socioeconomic characteristics is a unique situation, since there is a complex network of risk factors^(^ 19 ^)^, which can be mediated by internal and private issues of each subject^(^ 20 ^)^, which makes violence a multifaceted phenomenon.

When turning their attention to the pregnancy-puerperal cycle and the perpetuation of IPV, most women declared that they had not suffered violence during pregnancy. In contrast, in the postpartum period, IPV worsened, given the greater association between the types of violence suffered. In view of what was seen, two studies showed contrasting results: one found an incidence of violence in the postpartum period around 9.3%^(^ 19 ^)^, while the other^(^ 21 ^)^, even with a drop in violence compared to the gestational period, it was pointed out that 25.6% of the participants reported continuity in the puerperium. In this regard, it is noted that the puerperal woman is also exposed to IPV, regardless of whether the violence starts or continues in the puerperium.

In the practice of breastfeeding, it was evidenced that, at 180 days postpartum, only one woman was in EBF, and nine babies were weaned. These results show that abusive relationships can constitute barriers to the practice of BF, as observed in a study^(^ 22 ^)^ cross-sectional retrospective that analyzed 195,264 records and found that 11,766 women reported suffering some type of IPV during prenatal care, of which 36.3% did not breastfeed their children. Women who started breastfeeding and were in an IPV situation had an 18% increased chance of interrupting BF in the first eight weeks after delivery.

In the first thematic subcategory, it was evident that the partner was not recognized as part of the women’s SSN during the puerperium and breastfeeding process, a fact aggravated by the situation of IPV, since the bond was weakened. It is known that social support, when present and shared by the partner, has a positive impact on the woman’s esteem, helps with the emotional instabilities that may be present in this phase and contributes to the adaptation of the new social role. In addition, being present in the care of the baby reaffirms the affective bond and removes the woman from the position of sole responsible for the care, well-being, nutrition and development of the child^(^ 23 ^)^.

Interpersonal SSN was represented by women, especially mothers in-law, cousins, aunts and friends. This fact reaffirms that care during motherhood spreads symbolically between generations, especially among women, but the partner can also collaborate in the breastfeeding process^(^ 24 ^-^ 25 ^)^, different from the result observed in the present study. In addition, for some participants, the IPV situation accentuated a relational incompatibility with the family members. Therefore, it is noted that the breach of family precepts may come to contribute to SSN fragility^(^ 26 ^)^. In this regard, these weakened relationships promote a distance between these members and increase the chance of women remaining in situations of social vulnerability^(^ 27 ^)^.

In terms of functionality, interpersonal SSN was found in actions ranging from advices to helping to shelter women in relatives’ homes. However, in some situations, this SSN also indirectly contributed to the woman remaining in a situation of violence, with attitudes rooted in the role of gender and power relations. This result corroborates the literature, since, socially and culturally, socialization still naturalizes differences in behavior according to gender and, thus, puts women as fragile and submissive to men, a fact that can be intensified by relations with the SSN^(^ 19 ^,^ 28 ^-^ 29 ^)^.

Still in this context, women who remained at the side of their aggressors were stigmatized, which directly impacts the attempt to disrupt the network, as it can increase the partner’s power over women^(^ 30 ^)^. The blaming, on the part of the interpersonal network, accentuated the feelings of shame and failure, as well as isolation for the women in this study, allowing the invisibility of IPV, the non-blaming of the aggressor, the male domination relationship and, therefore, the perpetuation of violent relationship^(^ 11 ^,^ 29 ^,^ 31 ^)^.

In BF practice, it was found that family members, especially grandmothers, friends and neighbors were present, especially in the initial difficulties with the BF. The greater the intergenerational bond, the greater the sociocultural influence for the inclusion of food in the baby’s diet. This fact contributed to the discontinuity of EBF and, in some situations, added to the context of IPV and contributed to early weaning. In this regard, meta-synthesis^(^ 9 ^)^ who assessed the knowledge, attitudes and practices of grandmothers related to the support offered in the practice of BF showed that they are central figures and influence daughters or daughters-in-law to breastfeed, by offering support, at the same time that they can promote the discontinuity of the act breastfeeding through contrary opinions and inadequate information. Therefore, it is necessary to understand that the network, mediated by the interpersonal context, can both promote support, well-being and changes, as well as the disarticulation of the individual and his ongoing internal processes^(^ 6 ^)^.

In the second subcategory, the institutional SSN corresponded to the services required by women. However, it was reduced when compared to the interpersonal network, and was configured by little protective actions in the face of the IPV and breastfeeding, without intersectoral action. This finding can be explained by the structuring of institutional networks, which, being secondary networks, are characterized by relationships dominated by law, unlike primary networks (interpersonal), formed by significant relationships, of reciprocity and trust^(^ 11 ^)^. Thus, if the “micro network” is classified as the family network, the “macro network” is the one that includes the action of the community and society under the individual^(^ 6 ^)^.

In this study, institutional SSN was composed of the health, public security, judicial and NGO sectors. In the context of violence, the search for institutions that make up the network is called, in the literature, as a critical route^(^ 32 ^)^, since non-integrality and transversality culminate in the fragility of services and hinder conflict resolution, thus compromising the quality of care^(^ 32 ^-^ 33 ^)^.

Regarding the functionality of the institutional network, fragmented and low-resolution actions were observed, characterized by assistance to women through referrals. These actions, in addition to weakening the integrality of care, perpetuate the cycle of violence, since the woman does not feel welcomed and supported by the services^(^ 30 ^,^ 32 ^-^ 33 ^)^ given the understanding that IPV is something to be resolved within the family mainstay. Thus, attention is hardly configured as a resolving action^(^ 29 ^)^.

The same fragmented actions were observed in relation to the practice of breastfeeding, through which there was a predominance of assistance without qualified listening and without individualization of care. In order to change this model of care, new perspectives of care must be proposed, in order to facilitate meeting the demands. In the set of these actions, the permanent education of the professionals and the collaborative and interprofessional actions stand out, which provide a care more consistent with the real needs of women^(^ 34 ^)^.

Something significantly symbolic in this study was the approach of IPV and BF separately and as non-associated events, a fact that did not allow networks to provide functionality and meaning for these participants. In this sense, it appears that it is necessary to give greater visibility to the interface of violence and practices in breastfeeding, in order to ratify the merit of this study, as well as to justify the development of others on the subject at issue.

Nevertheless, in view of the submitted results, the importance of understanding the SSN as a dynamic thing is highlighted, that is, in which both - SSN and the individual - interact, sometimes in order to complement each other, sometimes to repel and, for therefore with the possibility of conflict. Thus, not all SSN can be totally beneficial or harmful, but they are built according to the context in which the subject is inserted, always aiming at the perspective of protection, which is why an individual without SSN tends to be more fragile and isolated^(^ 6 ^)^.

The greatest advancement of this study for scientific knowledge is linked to the fact that it understands that the IPV significantly interferes in the BF practice. In addition, it was possible to observe that both interpersonal and institutional SSN were not enough to change the trajectory of breastfeeding in situations of IPV. However, the main limitation of the study is related to the specificities of the participants, which can make it difficult to generalize these results to other contexts. On the other hand, the data presented here give rise to reflections on situations posed in society as complex phenomena, such as violence and breastfeeding. In this sense, for the production of scientific knowledge, understood, in this perspective, as unfinished and without the intention of producing absolute truths, since the singularities of the phenomena also depend on the social, political, economic and structural contexts in which the individuals are inserted.

## Conclusion

According to the meanings attributed by women in a situation of IPV and AM in relation to the SSN, the interpersonal SSN was more significant than the institutional network, both in the IPV and in the BF practice. In addition, both networks had a similar pattern of fragmentation of care with regard to the phenomena of violence and breastfeeding. Therefore, these phenomena were managed by them in isolation and not as associated events, that is, in a context in which violence could interfere in breastfeeding practice. In the actions, the interpersonal support network influenced BF practice through counseling or helping with household chores and baby care, which favored breastfeeding continuity. However, intergenerational care, marked by sociocultural beliefs, negatively interfered with breastfeeding.
